# The effect of an integrated care intervention of multidisciplinary mental health treatment and employment services for trauma-affected refugees: study protocol for a randomised controlled trial

**DOI:** 10.1186/s13063-022-06774-z

**Published:** 2022-10-08

**Authors:** Maja Bruhn, Henriette Laugesen, Matilde Kromann-Larsen, Cathrine Selnes Trevino, Lene Eplov, Carsten Hjorthøj, Jessica Carlsson

**Affiliations:** 1 Competence Centre for Transcultural Psychiatry, Mental Health Centre Ballerup, Mental Health Services of the Capital Region of Denmark, Ballerup, Denmark; 2 The Norwegian National Centre for Ageing and Health, Vestfold Hospital Trust, Tønsberg, Norway; 3 Department of Geriatric Medicine, Oslo University Hospital, Oslo, Norway; 4 Copenhagen Research Centre for Mental Health - CORE, Mental Health Centre Copenhagen, Gentofte, Denmark; 5 Department of Public Health, Section of Epidemiology, University of Copenhagen, Copenhagen, Denmark; 6 Department of Clinical Medicine, University of Copenhagen, Copenhagen, Denmark

**Keywords:** Refugee, Trauma, Post-traumatic stress disorder, PTSD, Cross-sectoral, Integrated care, Post-migration stressors, Ongoing stressors, Randomised controlled trial

## Abstract

**Background:**

The complexity of past trauma and ongoing post-migration stressors challenges the existing mental health treatment for trauma-affected refugees. Therefore, interventions are needed to accommodate these complex challenges in mental health treatment. This study examines the effect of an add-on integrated care intervention compared to treatment as usual (TAU) for trauma-affected refugees in a randomised controlled trial (RCT).

**Methods:**

The study is carried out at a Danish outpatient clinic and will include 197 treatment-seeking refugees with post-traumatic stress disorder (PTSD) who are unemployed and affiliated with municipal employment services. Mental health TAU comprises 10 sessions with a medical doctor (pharmacological treatment and psychoeducation) and 16–20 sessions with a psychologist (manual-based cognitive behavioural therapy) for a period of 8 to 12 months. The add-on intervention strengthens coordination between mental health treatment and employment interventions with three cross-sectoral collaborative meetings during the mental health treatment. The integrated care intervention draws attention to the bidirectional impact of mental health problems and post-migration stressors and focuses on cross-sectoral shared plans. The primary outcome is functioning, measured by WHODAS 2.0, the interviewer-administered 12-item version, with secondary outcomes measuring quality of life, mental health symptoms, and post-migration stressors.

**Discussion:**

The RCT is novel in intervention design for trauma-affected refugees and will bring forward new perspectives and knowledge of integrated care interventions for trauma-affected refugees. The integrated care intervention is expected to reduce post-migration stressors that negatively affect the treatment of trauma-related mental health problems, thereby improving preconditions for enhanced treatment outcomes. The intervention builds on existing practices in the Danish healthcare and employment sectors, which ensures high scalability and sustainability for future practices.

**Trial registration:**

ClinicalTrials.gov Identifier: NCT04244864, registered 28 January 2020. Protocol version: 17 September 2022, version 2.

**Supplementary Information:**

The online version contains supplementary material available at 10.1186/s13063-022-06774-z.

## Background

The number of refugees and forcibly displaced people have increased substantially in the past years, reaching 89.3 million forcibly displaced people and 27.1 million refugees in 2021 due to persecution, conflict, violence, or human rights violations [1]. The refugee experience is characterised by prolonged and repeated pre-migration traumas related to war and persecution and additional peri-migration traumas associated with the long and dangerous journeys to the country of safety. Thus, the trauma history of refugees generally differs from other trauma-affected populations with single or few traumatic events [2].

Trauma-affected refugees are at high risk of developing mental health problems, with an estimated prevalence of PTSD and depression among displaced and conflict-affected populations at approximately 30 % [2]. PTSD in refugees often turns into a more chronic disorder compared to other PTSD populations and with high rates of comorbidity [3, 4]. A meta-analysis has indicated that depression and anxiety disorders are twice as likely in refugees as in economic migrants [5].

In addition to the pre and peri-migration traumas, refugees are also subject to various post-migration stressors that can impact their mental health [6–8]. Common post-migration stressors are unemployment, poor finances and accommodation, language barriers, insecure residency status, limited social network, and worries about family living in unsafe conditions [6, 9, 10]. Some post-migration stressors are specifically related to the life circumstances of forced migration, such as changes in socio-economic status, language barriers, and family separation. Other stressors, such as unemployment and poor finances, are not limited to the refugee population [8, 11]. In the past years, the role of post-migration stressors has received increasing attention. A combination of pre, peri, and post-migration factors determines the rates and course of PTSD and other mental health problems [10, 12, 13]. While research demonstrates that post-migration stressors affect psychological functioning, how these stressors interact with treatment remains unclear [11].

Two meta-analyses found that psychosocial interventions improve PTSD and depressive and anxiety symptoms for refugees and asylum-seekers resettled in high-income countries [14, 15]. Both studies stress that the quality of evidence for their findings is moderate as the studies included are small with limited methodological quality. Five randomised controlled trials (RCTs) carried out at the mental health clinic of this trial protocol have investigated the outcomes of different treatment modalities for trauma-affected refugees (psychotherapy, psychopharmacological treatment, physical activity, and sleep disturbance targeted therapy) [16–20]. All five studies found a limited treatment effect on PTSD symptoms, which could be explained by the often chronic state of PTSD in refugees. Still, results also indicated that post-migration stressors such as unemployment could negatively predict treatment outcomes [21].

The interventions in the meta-analyses described above predominantly consist of trauma-focused therapies rather than interventions dealing with post-migration stressors. In a systematic review of psychosocial interventions for trauma-affected refugees, the authors highlight a substantial gap in our understanding of supporting refugees and asylum seekers with complex difficulties [22]. Further research is needed as refugee mental and physical health needs are complex and include concerns far beyond trauma-related symptomatology. The complexity includes the impact of traumatic events, post-migration stressors, and daily hassles among refugees. Interventions incorporating different modalities, such as social support and psychoeducation, in conjunction with trauma-specific techniques and attendance to post-migration and daily stressors generally lack scientific testing [11, 23]. A study found post-migration stressors to impact almost 40% of mental health treatment sessions with medical personnel [24]. Some suggest that post-migration stressors are prioritised as the initial focus of mental health treatment for refugees [25]. In line with this are recommendations for adding psychosocial interventions to multidisciplinary treatment to strengthen the social network, speed up the integration process, and find adequate employment [26].

In Denmark, there is a clear sectoral division of services, where social affairs are under the responsibility of the municipal social services sector, whereas mental health treatment lies in the regional health sector. The public employment services are located in the municipal job centres with the management of employment efforts, placement and active support for unemployed persons, as well as clarification of work capacity and nomination for disability pension [27]. The job centres are also responsible for managing cash benefits such as social assistance. An employment case counsellor is assigned when a person is affiliated with a job centre. The sectoral division and the lack of cross-sectoral collaboration specifically challenge the coherence of interventions and sector transitions for trauma-affected refugees [28]. Refugees with complex challenges such as unemployment and mental health problems need holistic, interdisciplinary interventions with coordinated and broad-spectrum support [29]. A review on the effect of cross-sectoral interventions for refugees found that previous research studies are few and of poor quality and concluded a great need for further research [30]. The authors suggest improving coordination between existing programmes, thereby gaining increased scalability of interventions, and pose that treatment of trauma-affected refugees should be specialised and integrated in a multi-layered and multi-sectoral care system.

In summary, the complexity of past trauma and ongoing post-migration stressors challenges the existing mental health treatment for trauma-affected refugees. Therefore, interventions are needed to accommodate these complex challenges in mental health treatment. This research gap led to the present trial.

### Research objectives and hypotheses

Based on the absence of relevant or conclusive data on integrated care interventions for trauma-affected refugees, we designed the present study to address post-migration stressors closely integrated in treatment for trauma-related mental health problems. This study aims to examine an add-on integrated care intervention of multidisciplinary mental health treatment and employment services for unemployed refugees with PTSD in a RCT.

The objectives are:

1. To investigate the treatment effect of an add-on multidisciplinary integrated care intervention on outcomes of functioning, quality of life, mental health symptoms, and level of post-migration stressors compared to TAU.
2. To examine the effect of treatment at 6-month follow-up on functioning, quality of life, and mental health symptoms, as well as job centre affiliation, labour market attachment and income at 12-months follow-up.

Based on previous research, we present the following hypotheses:

1. An integrated care intervention with treatment of trauma-related mental health problems and a focus on relieving post-migration stressors will cause improved treatment outcomes on functioning, quality of life, mental health symptoms, and levels of post-migrations stressors compared to TAU.
2. The intervention group will reveal better treatment outcomes at follow-up, including a speeded clarification process in the job centre compared to the TAU group due to improved cross-sectoral coordination and focus on post-migration stressors and unemployment in relation to mental health problems.

## Methods

### Trial design

The research study consists of a mixed method quantitative and qualitative study. A cost-utility analysis is also linked to the trial. This study protocol focuses on the quantitative methods of the RCT, while the qualitative study and cost-utility analysis are not further described in this protocol. The RCT design is a clinical pragmatic randomised controlled parallel-group two-arm superiority trial with a 1:1 allocation ratio. Approximately 197 patients will be included and randomised into the two groups: TAU and add-on of the integrated care intervention.

#### Participants and study setting

The study is carried out at the Competence Centre for Transcultural Psychiatry (CTP), a tertiary mental health service outpatient clinic in the Capital Region of Denmark. The clinic was established in 2008, and since 2009, the clinic has systematically carried out research programmes parallel with the treatment [31]. The target group of the clinic is treatment-seeking refugees with trauma-related mental health problems, as well as ethnic minorities with particularly complex mental health problems. Patients can be referred to the CTP by any MD, often their general practitioner (GP), a private practising psychiatrist, or an MD from mental health or somatic hospital. A senior psychiatrist at the CTP assesses all referrals, and based on the referral, patients are invited for an initial assessment by an MD. If it is clear from the referral that the patient does not belong to the clinic's target group, the patient is not invited for an assessment. This is the general clinical practice and has nothing to do with the trial.

The cross-sectoral collaboration in the intervention comprises the CTP and five municipalities in the Capital Region of Denmark: Copenhagen, Frederikssund, Gladsaxe, Lyngby-Taarbæk, and Hillerød. Data are exclusively collected at the CTP, and the municipalities are not involved in data collection and processing of the RCT.

Table 1 lists the full inclusion and exclusion criteria.

**Table 1:** Inclusion and exclusion criteria of the randomised controlled trial

Inclusion criteria	Exclusion criteria
• Adults (18 years or older) • Refugees or persons who have been family reunified with a refugee • PTSD pursuant to the ICD-10 research criteria • Psychological trauma experienced outside Denmark in the anamnesis^a^ • Home address in one of the five collaborating municipalities^b^ • Unemployed and assigned to a job centre^c^ in a collaborating municipality • Signed informed consent	• Severe psychotic disorder (defined as patients with an ICD-10 diagnosis F2x and F30.1-F31.9). Participants are excluded only if the psychotic experiences are assessed to be part of an independent psychotic disorder and not part of a severe PTSD and/or depression • Dependence syndrome of drugs or alcohol: active dependence and use (F1x.24-F1x.26)

^a^Trauma is imprisonment or detention with torture (according to the UN definition of torture) or acts of cruel, inhuman, and degrading treatment or punishment. Trauma can also be organised violence, long-term political persecution and harassment, or war and civil war experiences

^b^Municipality of Copenhagen, Frederikssund, Gladsaxe, Lyngby-Taarbæk, and Hillerød

^c^This includes patients receiving cash benefits of various types (e.g. social assistance, integration allowance). Disability pension and sickness benefit are not included

Five randomised controlled trials aiming to increase knowledge on the treatment of trauma-affected refugees been carried out at the CTP [16–20]. The trauma-affected refugees referred to the CTP derive from various countries, the majority originating from Syria, Iraq, Afghanistan, Iran, and Lebanon. In the previous RCTs, the mean duration of stay in Denmark is 13–15 years at the time of referral to the CTP. Approximately 40% of the patients have experienced torture. Comorbidity of depression varies from 70 to over 95% in the previous RCTs. Many of the patients have poor language proficiency in Danish and English. Danish interpreter service in sessions is required for 60-70% of the patients. About 90% of the patients have no current affiliation with the labour market, and half have no work experience in Denmark [17–20].

#### Initial assessment

An initial assessment by an MD is scheduled for all referred patients in the CTP target group. The assessment content is not specific to this trial but applies to all initial assessments at the CTP. The initial assessment consists of two to three sessions of approximately 45 min. It includes recording the trauma history, the migration process, social situation, somatic and psychiatric medical history, and a clinical and diagnostic assessment. Standardised diagnostic tools such as part of Schedules for Clinical Assessment in Neuropsychiatry (SCAN) and the ICD-10 research criteria are applied in the interview. PTSD assessment is supported by the PTSD part of the International Trauma Interview (ITI), which is under validation as a diagnostic measure of PTSD for ICD-11 [32, 33]. Furthermore, baseline outcome measures are obtained as described below.

All patients referred to the clinic who are interested in starting treatment and conform to the eligibility criteria are invited to participate in the study. Patients providing informed consent to participate to the MD are randomised after the initial assessment to either TAU or add-on intervention group.

#### The treatment as usual and add-on integrated care intervention

**Fig. 1:**
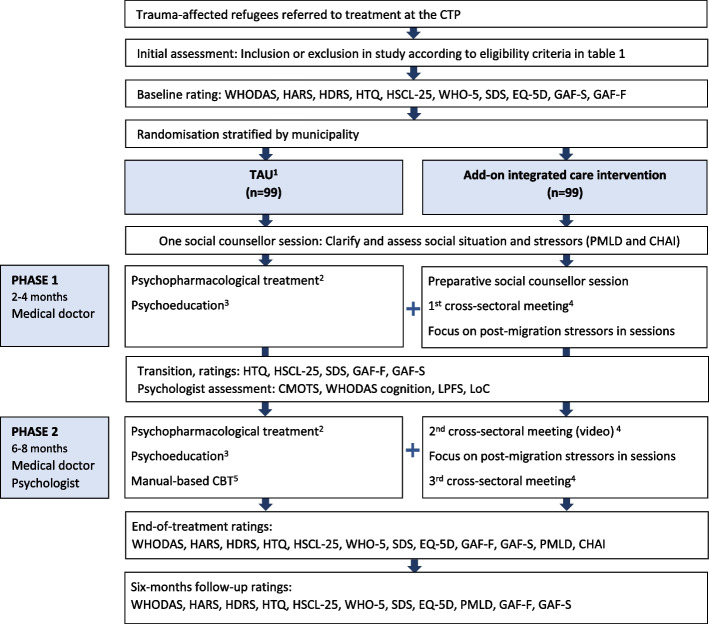
Flow diagram of interventions and ratings

The treatment and data collection will follow the SPIRIT statement [34]. Please see Fig 1.

#### Treatment as usual

The TAU offered to trauma-affected refugees at the CTP is eight to 12 months of multidisciplinary treatment comprising: 10 sessions with an MD, 16–21 sessions with a psychologist, and one session with a social counsellor. The treatment is divided into phases: phase 1 (2 to 4 months), weekly sessions with an MD (six sessions) and one session with a social counsellor, and phase 2 (6 to 8 months), monthly sessions with an MD (four sessions) and weekly sessions with a psychologist (16–21 sessions). Sessions are a standard of 45 min. Manuals are used in sessions with MDs and psychologists to establish shared standard procedures.

#### Sessions with the MD in treatment as usual

Focus in sessions is predominantly on pharmacological treatment and psychoeducation in an individually adjusted course. Relevant topics are addressed, such as knowledge of trauma reactions, PTSD, depression, anxiety), pain, sleep, concentration, memory, exercise, lifestyle, and relaxation techniques. Pharmacological treatment follows an algorithm in the MD's treatment manual. According to this manual algorithm, sertraline is the first choice antidepressant and venlafaxine the second choice. Mianserin or mirtazapine are the first choices for severe sleep disturbances.

#### Sessions with the psychologist in treatment as usual

Within the first couple of psychologist sessions, the psychologist assesses reflective functioning and plans the individual course of treatment. The assessment tools are described further in the “Psychologist assessments” section. The psychologist can apply various combinations of cognitive behavioural therapy, e.g. trauma-focused cognitive behavioural therapy (TF-CBT), acceptance and commitment therapy (ACT), mindfulness, stress management (SM), and cognitive restructuring (CR). A manual has been developed based on cognitive therapy with adaption to the target group. We refer to this as manual-based cognitive behavioural therapy (manual-based CBT).

#### Sessions with the social counsellor in treatment as usual

At the beginning of the treatment course, all patients are offered one session with a social counsellor to clarify and assess the social situation. Patients can receive additional assistance from a social counsellor during the treatment if needed. This offer is individualised. Typically, such sessions are scheduled if patients experience a very pressing and acute social problem, e.g. loss of citizenship, loss of housing, or financial aid. Thus, most patients receive only one or two sessions during the entire course of treatment [24]. The TAU does not include a systematic focus on managing and addressing post-migration stressors.

#### Cross-sectoral collaboration in treatment as usual

Cross-sectoral collaboration between the CTP and the municipalities in TAU will continue in its current form. If a patient enrolling in treatment is associated with a job centre, the CTP social counsellor sends a letter or e-mail to the municipal employment case counsellor to inform about the patient starting mental health treatment. The same applies to ending treatment, where a letter of information is also sent. Both parties can request a cross-sectoral collaborative meeting under the Cooperation Agreement [35]. These cross-sectoral meetings are not systematically planned and are relatively rare in practice.

Collaboration with the patient’s general practitioner (GP) in the primary sector includes the MDs sending a letter to the GP at the beginning and end of treatment and as required during the treatment.

#### Add-on integrated care intervention

Overall, the add-on intervention strengthens coordination between mental health treatment and employment interventions through three cross-sectoral collaborative meetings during the mental health treatment. The integrated care intervention draws attention to the bidirectional impact of mental health problems and post-migration stressors and focuses on cross-sectoral shared plans.

#### The tools implemented in the add-on intervention

A central tool used is the Action Plan to ensure a systematic approach to addressing and managing stressors as part of the treatment. The Action Plan is a comprehensive patient-centred plan to be used across sectors. In the Action Plan, stressors and challenges related to seven aspects are systematically assessed: employment/education, economy, family, leisure activities and social life, accommodation, physical and mental health, and other stressors. For each stressor the patient experiences, the Action Plan includes a specific plan for managing the patient’s challenges, including short- and long-term goals and specific actions to achieve these goals. The Action Plan is a dynamic document that is revised continuously during the treatment. The Action Plan is a central tool for all collaborative meetings, preparative sessions for the collaborative meetings, and the treatment sessions. The Action Plan must be a valuable tool for both the patient and professionals. The use of the Action Plan at collaborative meetings and treatment sessions is documented in the case report form (CRF) after each session. The Action Plan derives from the “Collaborative Meeting Instrument” (*Netværksmøde-skabelon*), which was developed, tested, and evaluated in a separate project in the Capital Region of Denmark [36]. We adapted this instrument to our patient group and tested it for acceptability and meaningfulness in a pilot study before inclusion. The structural implementation of the Action Plan in all aspects of the treatment course is, to our knowledge, unique for this study.

#### Integration of the cross-sectoral collaboration in the add-on intervention

The cross-sectoral part of the add-on intervention holds a structure of three collaborative meetings between the patient, the employment case counsellor from the job centre, and clinicians from the CTP. Counsellors from other municipal social care sections can attend when relevant. People from the patient’s network are also invited to participate, e.g. a family member or mentor, if this is relevant to the patient. The collaborative meetings aim to share knowledge across sectors, provide a tangible plan for dealing with post-migration stressors, and coordinate municipal social interventions and mental health treatment. Attention is brought to empowering the patients during these meetings and facilitating the ownership of the Action Plan.

Before the collaborative meetings, the patient is offered a preparative session with a social counsellor at the CTP, where the patient's current post-migration stressors are addressed and documented in the Action Plan.

The initial collaborative meeting takes place within the first 2 months of mental health treatment. At the meeting, the first draft of the Action Plan is reviewed and revised. Status and the current plan for mental health treatment and employment and social care interventions are mutually shared. The interventions in the municipality and the mental health treatment at the CTP are coordinated following the patient's resources. Meeting participants are the patient, the MD, the CTP social counsellor, and the employment case counsellor.

The halfway collaborative meeting takes place after approximately eight sessions with the psychologist. This meeting seeks to follow-up on the Action Plan, revise actions and goals if required, and secure that interventions are continuously coordinated across sectors. Meeting participants are the patient, the psychologist or the MD, the CTP social counsellor, and the employment case counsellor.

The concluding collaborative meeting will primarily focus on the sector transition, which involves planning the subsequent period on how to support the patient in the job centre after ending treatment. Meeting participants are the patient, the psychologist and the MD, the CTP social counsellor, and the employment case counsellor.

The initial and concluding collaborative meetings are highly prioritised as physical attendance at the CTP. Suppose a physical meeting is not possible, e.g. due to COVID-19 restrictions. In that case, it is arranged for the employment case counsellor to attend virtually while the other participants are in physical attendance at the CTP. The halfway meeting will most often be in this hybrid format. If the patient does not show up for the collaborative meeting and has not cancelled in advance, the employment case counsellor and the CTP clinicians will complete the meeting without the patient (if the patient has given oral consent for this in the preparatory session). This is not optimal but is considered necessary due to the daily clinical setting. If the patient cancels in advance, the meeting is rescheduled.

In-between the collaborative meetings, the involved parties are in ongoing contact over e-mail and phone. The CTP social counsellors are responsible for booking and coordinating the collaborative meetings. Patients are offered additional individual sessions with the CTP social counsellor when needed.

#### Post-migration stressors in the treatment sessions in the add-on intervention

During treatment sessions, the clinicians integrate a systematic focus on the actions agreed on in the Action Plan and implement strategies for coping with these post-migration stressors. All clinicians follow professional group-specific manuals for the add-on intervention. The manuals provide detailed information on options for implementing a focus on post-migration stressors in treatment sessions. An essential theme during sessions with all clinicians is the patient's empowerment. In cases where post-migration stressors are unalterable, e.g. due to national legislation, there will be a thorough clarifying process of their social situation to enhance empowerment and provide the patients with an overview and understanding of their situation. Common unalterable stressors are often related to family living in unsafe conditions and residence permit.

#### The organisation of the cross-sectoral collaboration in the add-on intervention

Performing cross-sectoral studies is well-known to include several challenges. We have accounted for some of the most prominent challenges by, e.g. inviting representatives from the collaborating municipalities to five joint meetings in 2018 during the development of the intervention, thereby securing ownership of the intervention for all parties involved. All five municipalities have agreed on the content of the cross-sectoral part of the intervention and have signed a legally binding collaboration contract. To ensure a well-functioning cross-sectoral practice, a specially designated intervention manager from the CTP (a social counsellor) and a key person from each municipality are responsible for the overall cross-sectoral collaboration.

### Outcome

**Fig. 2:**
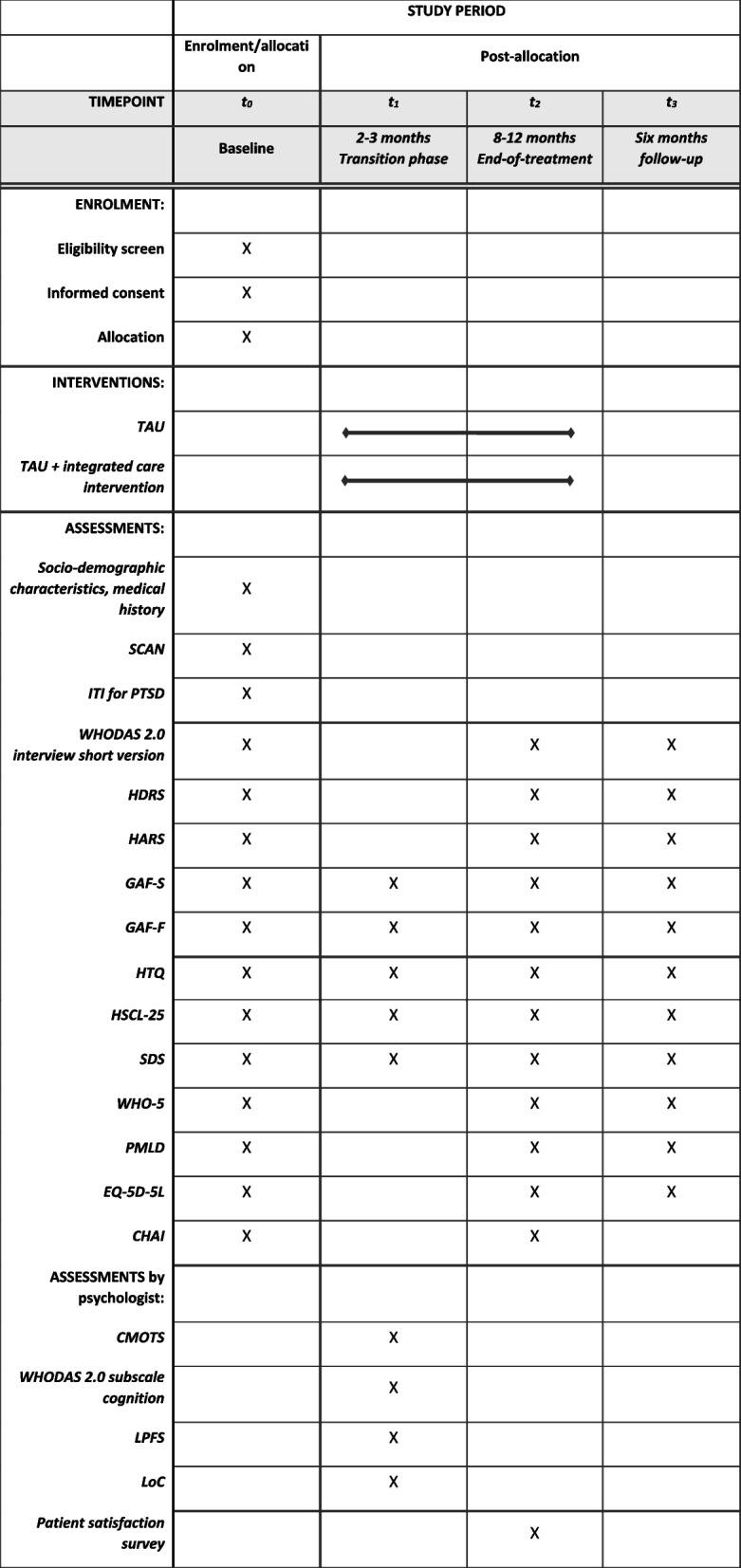
Schedule of enrolment, interventions, and assessments

Evaluation of treatment outcome in the study is measured with observer rating scales and self-administered ratings (please see Fig. 2). Time points of measures are baseline, between phases 1 and 2 (at 2–3 months), end-of-treatment (at 8–12 months), and 6 months follow-up. The rating scales between phases 1 and 2 serve as baseline measures in research sub-studies on psychotherapy. The clinicians are thoroughly trained in using the observer ratings and interviews. The selected ratings are used frequently in clinical studies on PTSD for refugees and other patient groups and are validated in several languages and cultural settings. Because of the chronic state of PTSD in many refugees, treatment is often primarily targeted at enhancing functioning and quality of life, which is why symptom-related outcome measures do not necessarily reflect this group’s clinically relevant treatment outcome [16–18]. This is the rationale for selecting a functioning rating scale as the primary outcome measure. We assessed The World Health Organization Disability Assessment Schedule 2.0 (WHODAS 2.0), the interviewer-administered 12-item version, as the most appropriate primary outcome measure for the study population due to high internal consistency and test-retest reliability in cross-cultural settings. Additionally, a study found WHODAS 2.0 to serve as a proxy measure for mental health status in refugees [37].

### Primary outcome measure

*The World Health Organization Disability Assessment Schedule 2.0 (WHODAS 2.0)* [38], the interviewer-administered 12-item version, measures health and disability across cultures in six core life domains of cognition/communication, mobility, self-care, interpersonal interaction, life activities, and participation in society. The items are rated on a five-point Likert scale of functional impairment (0 = none; 1 = mild; 2 = moderate; 3 = severe; 4 = extreme). The WHODAS has been widely used across epidemiological surveys worldwide, proving sound internal consistency (Cronbach *α* = 0.87 for the entire scale) and test-retest reliability (intraclass correlation coefficients ranging from 0.69 to 0.89 across items). The MDs perform the WHODAS interviews group-blinded at baseline (before randomisation) and unblinded at end-of-treatment. At 6-month follow-up, the WHODAS interview is carried out group-blinded by a trained medical student.

### Secondary outcome measures

#### Observer rating scales

*Hamilton depression and anxiety rating scales (HDRS and HARS)* [39, 40] are observer rating scales assessing depression and anxiety, respectively, in semi-structured interviews. The scales have been used widely in psychiatric research including for trauma-affected refugees. For HDRS, the 17-item version is used. A team of medical students perform the Hamilton interviews and take part in regular joint ratings to ensure high quality and interrater reliability. The Hamilton assessors are blinded to the intervention arms and time points of assessments.

*Global Assessment of Functioning*—*Symptoms (GAF-S) and Functioning (GAF-F)* [41] are numeric observer rating scales used to assess the degree of social and psychological functioning in adults. The MDs perform the WHODAS interviews group-blinded at baseline (before randomisation) and unblinded at end-of-treatment. At 6-month follow-up, the WHODAS interview is carried out group-blinded by a trained medical student.

#### Self-administered rating scales

The self-rating scales are available in Danish, English, Arabic, and Farsi. Due to the multi-cultural setting, patients that do not read these languages will have the questionnaires read aloud and translated by the interpreter.

The following scales are applied.

*The Harvard Trauma Questionnaire (HTQ)* [42] is an internationally applied and thoroughly validated self-administered rating scale assessing the severity of PTSD symptoms. The first 16 questions of the HTQ, Part IV (symptoms part), are used to monitor PTSD symptoms. These 16 questions cover all PTSD criteria in accordance with ICD-10 and DSM-IV.

*Hopkins Symptom Check List (HSCL-25)* [43] is an internationally applied and thoroughly validated self-administered rating scale assessing the severity of anxiety and depression symptoms. This is a short version of the Symptom Checklist-90 (SCL-90). It consists of 25 questions, 10 regarding anxiety, and 15 regarding depression.

*WHO-5* [44] is a self-administered questionnaire evaluating quality of life, consisting of five questions with every six possible answers. The questionnaire has been used to assess the quality of life in several psychiatric diagnostic groups. In addition, the scale has been used to assess overall treatment effects in psychiatry.

*Sheehan Disability Scale (SDS)* [45] is a self-administered rating scale measuring functional impairment related to family, work, and social network using three visual analogue scales from 0 to 10. Evaluation of the scale has shown that it is sensitive to treatment effects on mental health.

### Explorative outcome measures

#### Self-administered rating scales

The self-rating scales are available in Danish, English, Arabic, and Farsi (except CHAI).

*Post-migration Living Difficulties Check List (PMLD)* [6] is a self-administered rating scale examining the extent to which post-migration challenges were a problem for the patient, originally over the past 12 months. PMLD was developed for refugees and asylum seekers in Australia. The developers recommend always adapting the scale to the context it is applied to. The version used for our study is adapted to the Danish context as a 17-item scale. The adaption includes inspiration from items from a Swiss and Swedish version of PMLD, which are culturally comparable to a Danish context on several parameters [46, 47]. Due to the nature of the present RCT, the timeframe is reduced to the past one month. Items are rated on a five-point Likert scale (0 = not a problem to 4 = very serious problem). Items scored at least 2 (“moderately serious problem”) are considered positive responses, yielding a total count of living difficulties. The PMLD scale has been identified as a predictor of mental health among displaced populations [6, 46, 47].

*European Quality of Life (EQ-5D-5L)* [48, 49] is a self-administered questionnaire on health status comprising five dimensions: mobility, self-care, usual activities, pain/discomfort, and anxiety/depression. Each dimension has five levels of problem severity. In addition, EQ-5D includes EQ VAS, which records the patient’s self-rated health on a vertical visual analogue scale, where the endpoints are labelled ‘The best health you can imagine’ and ‘The worst health you can imagine’. The VAS can be used as a quantitative measure of health outcome that reflects the patient’s own judgement. EQ-5D is also suitable for cost-utility analyses.

*Consumer Health Activation Index (CHAI)* [50] is a 10-item self-rating scale assessing patient activation based on five key domains: knowledge, self-efficacy, motivation and beliefs, actions, and internal locus of control. The questionnaire is rated on a six-point Likert scale ranging from ‘Strongly disagree’ to ‘Strongly agree’. Afterwards, the scores are transformed into a theoretical value from 0 to 100, with higher scores representing a stronger involvement in the treatment and confidence in the ability to take care of one's health and health treatment. Higher CHAI scores have been associated with fewer depressive and anxiety symptoms. CHAI has been found valid and reliable with good internal consistency. It has not previously been used on trauma-affected refugees.

#### Psychologist assessments

In addition to the outcome measures described above, the psychologists use the following assessment tools in the first sessions of the psychologist treatment course before initiating psychotherapy.

*Client Motivation for Therapy Scale (CMOTS)* [51] is used as a semi-structured interview assessing motivation for therapy. It consists of 24 questions, with every five possible answers.

*The World Health Organization Disability Assessment Schedule (WHODAS 2.0)* [38] subscale on cognition is an interviewer rating scale with six questions used to assess concentration, memory, problem-solving, learning, and communication.

*Level of Personality Functioning Scale (LPFS)* [52] is an observer rating scale used to assess personality-related function with reference to the normal personality. In addition to the initial assessment, this is also applied after eight psychologist sessions.

*Locus of Control (LoC)* [53] is used in an adapted scale to assess the degree to which people believe they have control over their own lives*.*

#### Register data

In addition to data collected at the CTP, benefit and income status are retrieved from the Danish DREAM database and the electronic income register from baseline to 12-month follow-up [54]. The register data explore income and job centre affiliation changes in the period. As an explorative outcome, register data on the consumption of health care services from 12 months before treatment to 12-months follow-up are also retrieved.

#### Evaluation and follow-up

All courses of treatment are completed with an evaluation interview with the patient and the multidisciplinary clinician team. At the end of treatment, the patient completes a questionnaire on treatment satisfaction. The satisfaction survey is five questions developed at the CTP. Six months after the end of treatment, all study participants are invited to participate in a follow-up interview that includes interviews and ratings. A medical student performs the follow-up interviews. If patients do not show up for follow-up interviews, the interviewer calls them on the phone and tries to reschedule.

#### Interpreters

Interpreters are used in treatment sessions and are present at ratings as required. The interpreters are all affiliated with the clinic and are experienced in interpreting questionnaires, psychotherapy, and mental health consultations. The patients do not pay any service charge for using interpreters.

#### Randomisation and blinding

Participants are randomised after a total of two to three-hour initial assessment with the MD in accordance with the inclusion and exclusion criteria. The allocation ratio is 1:1, and the randomisation is stratified by municipality. The randomisation and stratification are carried out in REDCap (Research Electronic Data Capture). The REDCap allocation sequence is generated by a researcher not directly involved in the trial and is unavailable to the investigator, sponsor, and clinicians.

Blinding patients and practitioners are not assessed as appropriate due to the different nature of the treatment interventions. The WHODAS interview, GAF-F, and GAF-S are performed group-blinded at baseline (before randomisation) and follow-up, but unblinded at end-of-treatment. The interviewers performing the Hamilton ratings (depression and anxiety) are blinded to both intervention group and time point of assessment at baseline, end-of-treatment, and follow-up. Data assessment and analysis, as well as conclusions, are performed blinded.

#### Representativity, trial fidelity, and variability

Patients are not selected based on more specific criteria than elsewhere in the treatment system. They, therefore, represent the population at other clinics treating trauma-related mental health problems in refugees. The results can thus be generalised to other corresponding patient groups and are directly applicable in the treatment planning for this patient population.

To determine trial fidelity, patient attendance is registered. After each consultation with an MD, psychologist, or social counsellor, the topics addressed are registered, as well as the methods used during the consultation and whether the patient has completed exercises between sessions as planned. At each consultation with an MD, any changes in medication are also recorded.

If collaborative meetings are cancelled, rescheduling is attempted, but this may cause variation in the timing of meetings in the treatment course. This is registered in addition to content and participants in the respective collaborative meetings. The use of the Action Plan at collaborative meetings and treatment sessions is also registered.

All patients will follow the treatment described above as accurately as possible. Still, due to the pragmatic nature of the study, some variation in attendance and timing of meetings and sessions is expected.

#### Extraction criteria

Patients are withdrawn from the trial if a severe psychotic disorder (ICD-10 diagnosis F2x and F30.1-F31.9) or dependence syndrome of drugs or alcohol with active dependence and use (F1x.24-F1x.26) is diagnosed during the study. Patients who do not want to continue in the study are withdrawn.

#### Completers and drop-out of the study

Patients are considered to have completed treatment when attending a minimum of five MD sessions and 10 psychologist sessions. In addition, two collaborative meetings must be held during treatment for the intervention group to be considered complete. An exception of this completer criteria is if fewer collaborative meetings are held because of termination of job centre intervention, e.g. due to getting employed or acceptance for disability pension. All patients who drop out of treatment are asked about their reasons for this. These reasons and the time of the drop-out of treatment are registered.

#### Power calculation

A minimal clinically important difference score for the WHODAS 2.0 has not yet been established. It is difficult to find studies with populations comparable to the present study in the literature. Based on clinical experience and the sparse available literature, a conservative minimal clinically important difference was taken to be five scale points on WHODAS 2.0 12-item version, and within-groups SD was taken to be 10 scale points. With a power level of 80% and alpha of 0.05, we estimate a sample size of each group of 64 and a total of 128. The completion rate in the preceding randomised trials at the CTP was two thirds, and we, therefore, set the expected drop-out rate to 35% for this study. The investigators increased the number of patients included to 128 × (1/ (100%-35%)) and, consequently, estimated a total sample size of 197 patients. Inclusion stops when approximately 197 patients are included in the trial. In the case that a minimal clinically important difference for WHODAS 2.0 is established during the time frame of the trial, this will be considered in the analyses.

For the secondary outcome measures with an SD of 0.5, we have 80% power to detect a change of 0.21 on HTQ, 3.03 on HDRS, 3.77 on HARS, 0.25 on HSCL, 3 on SDS, 8 on WHO-5, 4.05 on GAF-F, and 2.85 on GAF-S.

#### Drop-out analysis

Drop-out analysis is based on the patients who show up at the pre-treatment assessment. The participants are compared to the patients excluded at the initial assessment on several dimensions to identify possible systematic selection bias. The participants included in the trial but who eventually drop out and do not complete the trial are analysed in an intention-to-treat analysis. In addition, completer analyses are carried out.

#### Monitoring, quality control, and steering committee

Quality control and quality assurance follows regular procedures as described in the Danish Executive Order on Good Clinical Practice (GCP). The previous RCTs at CTP has been under GCP monitoring, but the GCP Unit has assessed that GCP monitoring is not necessary for the current trial as CTP is assessed to have high-quality internal monitoring. The internal monitoring team are independent from the intervention and have no competing interests. When an MD recruits a participant and obtains written informed consent, the monitoring team afterwards check that it is filled out correctly. All clinicians fill out the CRF using REDCap, while the monitoring team supervises the REDCap entries and that outcome measures are collected correctly at the described time points. The monitoring team registers ratings in REDCap using double entry.

The Trial Steering Committee consists of the sponsor, the investigator, the researcher responsible for the qualitative study, and the social counsellor responsible for the cross-sectoral collaboration. The Trial Steering Committee meets every second month to ensure that the trial conforms to the protocol and are also available to the clinicians for day to day support and questions regarding the trial and participants. Additionally, two external associate professors, who are independent from the data collection, are continuously supervising the trial with yearly meetings with the Trial Steering Committee and will supervise the data analysis and interpretation of data.

#### Data processing

A detailed statistical analysis plan (SAP) is submitted to the study protocol before data collection ends.

The primary outcome variables are differences during the treatment course calculated as differences between baseline and end-of-treatment ratings. The differences between the two intervention groups can be measured with adjustment for baseline variables by ANCOVA/linear regression and with multiple imputations to handle missing data.

Six-month follow-up outcome variables will be analysed, corresponding to end-of-treatment. Benefit and income status are retrieved from the Danish DREAM database and the electronic income register from baseline to 12-months follow-up [54]. The DREAM database is administered by the Danish Agency for Labour Market and Recruitment and can be linked to different registers, including the Danish Income Register.

#### Publication of results

Positive as well as inconclusive or negative results will be published. We plan two publications for the RCT corresponding to the objectives of the study:

1. To investigate the treatment effect of an add-on multidisciplinary integrated care intervention on outcomes of functioning, quality of life, mental health symptoms, and level of post-migration stressors compared to TAU
2. To examine the effect of treatment at six-month follow-up on functioning, quality of life, and mental health symptoms, as well as job centre affiliation, labour market attachment and income at 12-months follow-up.

If the results cannot be published in a journal, they will be published at www.clinicaltrials.gov or www.clinicaltrialsregister.eu.

Trial results will be communicated to patients via e-mail.

The Vancouver rules for authorship are followed. There will be no use of professional writers.

## Discussion

This study protocol, The effect of an integrated care intervention of multidisciplinary mental health treatment and employment services for trauma-affected refugees: study protocol for a randomised controlled trial, is following the Standard Protocol Items: Recommendations for Interventional Trials (SPIRIT) 2013 statement for clinical trial protocols; please see Additional file 1.

The trial intervention focusing on post-migration stressors in close cross-sectoral collaboration will reduce the stress level that negatively influences the treatment of trauma-related mental health problems, thereby improving preconditions for enhanced treatment outcomes.

The post-migration stressors targeted and sought managed during the trial in the add-on intervention are the patients’ self-perceived stressors. Because all patients included in the trial are assigned to a job centre, meaning that they are all unemployed and receiving financial social benefits, these challenges are expected to attract attention in the intervention. The professional assessment of the impact of mental health challenges on functioning and vocational rehabilitation possibilities is a key element. Many patients will not have been employed for years—if ever in Denmark—and for some in this group, vocational rehabilitation is not considered realistic, not in short nor in the long term, because of their poor mental health and limited level of functioning. The close cross-sectoral collaboration and coordination also seek to speed up the clarification process in the job centre, which will especially benefit these patients and has been a central desire from representatives of the job centres when planning the intervention. We also expect stress related to accommodation and issues in the immediate family living in Denmark to be addressed and sought relieved in the cross-sectoral collaboration. We expect to be able to actively change conditions regarding the following stressors: Employment/education and thus finances, leisure activities, and physical and mental health. Accommodation and visa status are stressors that might be challenging to amend in the intervention as the current Danish legislation limits possibilities for changes of these stressors. The employment case counsellor is viewed as a coordinator of social interventions and is expected to involve other stakeholders, e.g. the social care services, when needed. It is a limitation to the intervention that other relevant stakeholders are not routinely attending the collaborative meetings.

Previous research has not found an association between the number of treatment sessions and outcome. Therefore, an improved outcome in the intervention group can be considered a result of the content of the intervention rather than the higher number of treatment sessions in the add-on intervention [20, 55].

A limitation of the trial is that neither the patients nor the clinicians are blinded to the treatment groups. The variety of outcome measures enables comparison of results with other trials on the same patient group. The WHODAS interview, GAF-F, and GAF-S are performed group-blinded at baseline (before randomisation) and follow-up, but unblinded at end-of-treatment. The self-ratings and unblinded observer outcome measures have been pragmatically selected to fit this trial into everyday clinical practice but are a clear limitation in the study. These outcome measures are supported by blinded assessors of Hamilton interviews for depression and anxiety at baseline, end-of-treatment, and follow-up, thereby reducing the risk of biased effect estimates. Performing intention-to-treat analysis will also reduce this risk. Analyses and conclusions are carried out blinded to limit interpretation bias.

The RCT is novel in intervention design for trauma-affected refugees and will bring forward new perspectives and knowledge of integrated care interventions for trauma-affected refugees. The intervention builds on existing practices in the Danish health care and employment services, which ensures high scalability and sustainability for future practices. In addition to the RCT presented in this study protocol, a qualitative study and cost-utility analysis are linked to the trial to strengthen the evaluation of the intervention in this complex field of research.

### Trial status

Screening of patients for the trial started on 3 February 2020. The first participant was recruited on 25 February 2000. Inclusion is expected to be completed in August 2023 and the last patient completing the intervention by August 2024.

## Supplementary Information

**Additional file 1.** SPIRIT 2013 Checklist: Recommended items to address in a clinical trial protocol and related documents^*^.

**Additional file 2.** CONSORT 2010 Flow Diagram.

## Data Availability

The research materials (treatment manuals, consent forms etc.) will be available upon request to the corresponding author. The final data set will be available to the investigator and sponsor. We disclose no contractual agreements limiting investigators’ access to the final trial dataset. The data supporting the findings of this study and statistical code are available from the corresponding author upon reasonable request within limits of the Danish Data Protection legislation, as is the full protocol. Any data required to support the protocol can be supplied on request.

## Abbreviations

ANOVA: Analysis of variance
CBT: Cognitive behavioural therapy
CHAI: Consumer Health Activation Index
CMOTS: Client Motivation for Therapy Scale
CRF: Case report form
CTP: The Competence Centre for Transcultural Psychiatry
DSM: Diagnostic and Statistical Manual of Mental Disorders
EQ-5D-5L: European Quality of Life - Five Dimensions - Five Levels
GAF-F: Global Assessment of Functioning—Functioning
GAF-S: Global Assessment of Functioning—Symptoms
GCP: Good Clinical Practice
GP: General practitioner
HARS: Hamilton Anxiety Rating Scale
HDRS: Hamilton Depression Rating Scale
HSCL-25: Hopkins Symptom Checklist-25
HTQ: Harvard Trauma Questionnaire
ICD-10: International Classification of Diseases, 10th Edition
ICD-11: International Classification of Diseases, 11th Edition
ITI: International Trauma Interview
LoC: Locus of control
LPFS: Level of Personality Functioning Scale
Manual-based CBT: Manual-based cognitive behavioural therapy
MD: Medical doctor
PMLD: Post-migration Living Difficulties Check List
PTSD: Posttraumatic stress disorder
REDCap: Research Electronic Data Capture
RCT: Randomised controlled trial
SAP: Statistical analysis plan
SCAN: Schedules for Clinical Assessment in Neuropsychiatry
SDS: Sheehan Disability Scale
TAU: Treatment as usual
WHO-5: Well-being Index-five
WHODAS 2.0: The World Health Organization Disability Assessment Schedule 2.0
