# Supplementary material for: The effect of an integrated care intervention of multidisciplinary mental health treatment and employment services for trauma-affected refugees: study protocol for a randomised controlled trial
Source: Trials. 2022 Oct 8;23:859. doi: 10.1186/s13063-022-06774-z (PMC9547630; doi:10.1186/s13063-022-06774-z)
Supplement: Supplementary file 2 — Additional file 2. CONSORT 2010 Flow Diagram. [file 13063_2022_6774_MOESM2_ESM.doc]

**
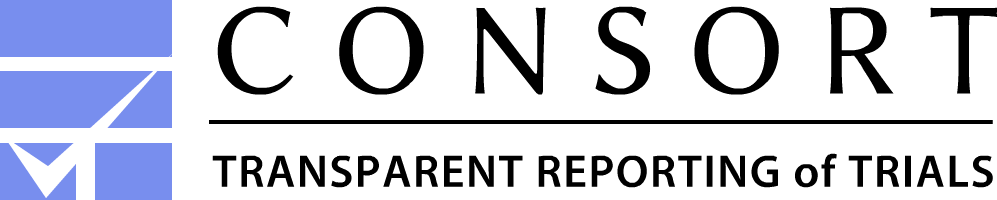
**

**CONSORT 2010 Flow Diagram**

**Allocation**

**Analysis**

**Follow-Up**

**6-months**

**Enrollment**

Assessed for eligibility (n= )

Excluded (n= )

♦  Not meeting inclusion criteria (n= )

♦  Declined to participate (n= )

♦  Other reasons (n= )

Analysed (n= )
♦ Excluded from analysis (give reasons) (n= )

Lost to follow-up (give reasons) (n= )

Discontinued intervention (give reasons) (n= )

Allocated to treatment as usual (n= 99)

♦ Received allocated intervention (n= )

♦ Did not receive allocated intervention (give reasons) (n= )

Lost to follow-up (give reasons) (n= )

Discontinued intervention (give reasons) (n= )

Allocated to TAU + add-on integrated care intervention (n= 99)

♦ Received allocated intervention (n= )

♦ Did not receive allocated intervention (give reasons) (n= )

Analysed (n= )
♦ Excluded from analysis (give reasons) (n= )

Randomized (n= 197)
